# Provider cost analysis supports results-based contracting out of maternal and newborn health services: an evidence-based policy perspective

**DOI:** 10.1186/1472-6963-14-459

**Published:** 2014-11-13

**Authors:** Peter Hatcher, Shiraz Shaikh, Hassan Fazli, Shehla Zaidi, Atif Riaz

**Affiliations:** Department of Community Health Sciences, Aga Khan University, Karachi, Pakistan; Women & Child Health Division, Aga Khan University, Karachi, Pakistan

**Keywords:** Contracting out, Provider cost, Maternal and newborn health

## Abstract

**Background:**

There is dearth of evidence on provider cost of contracted out services particularly for Maternal and Newborn Health (MNH). The evidence base is weak for policy makers to estimate resources required for scaling up contracting. This paper ascertains provider unit costs and expenditure distribution at contracted out government primary health centers to inform the development of optimal resource envelopes for contracting out MNH services.

**Methods:**

This is a case study of provider costs of MNH services at two government Rural Health Centers (RHCs) contracted out to a non-governmental organization in Pakistan. It reports on four selected Basic Emergency Obstetrical and Newborn Care (BEmONC) services provided in one RHC and six Comprehensive Emergency Obstetrical and Newborn Care (CEmONC) services in the other. Data were collected using staff interviews and record review to compile resource inputs and service volumes, and analyzed using the CORE Plus tool. Unit costs are based on actual costs of MNH services and are calculated for actual volumes in 2011 and for volumes projected to meet need with optimal resource inputs.

**Results:**

The unit costs per service for actual 2011 volumes at the BEmONC RHC were antenatal care (ANC) visit USD$ 18.78, normal delivery US$ 84.61, newborn care US$ 16.86 and a postnatal care (PNC) visit US$ 13.86; and at the CEmONC RHC were ANC visit US$ 45.50, Normal Delivery US$ 148.43, assisted delivery US$ 167.43, C-section US$ 183.34, Newborn Care US$ 41.07, and PNC visit US$ 27.34. The unit costs for the projected volumes needed were lower due to optimal utilization of resources. The percentage distribution of expenditures at both RHCs was largest for salaries of technical staff, followed by salaries of administrative staff, and then operating costs, medicines, medical and diagnostic supplies.

**Conclusions:**

The unit costs of MNH services at the two contracted out government rural facilities remain higher than is optimal, primarily due to underutilization. Provider cost analysis using standard treatment guideline (STG) based service costing frameworks should be applied across a number of health facilities to calculate the cost of services and guide development of evidence based resource envelopes and performance based contracting.

## Background

### Introduction

Resource allocation is one of the biggest challenges confronted by fragile health systems. Limited budgets are mostly allocated by government without costing the services to be provided. Existing literature has mainly focused on costing of disease specific public health interventions such as Tuberculosis and HIV [1], immunization programs [2], or specific services such as maternal health services [3, 4]. Although attempts have been made to estimate costs of scaling up primary healthcare services, costs of emerging reforms in health sectors of developing countries are not well captured. Contracting out of government health facilities to non-governmental organizations (NGOs) is one such reform initiative which has shown promise in improving access to primary healthcare services in some countries [5, 6]. However, little attention has been paid to costing of contracted out services, particularly for Maternal and Newborn Health (MNH) services, and the evidence base is weak for policy makers to estimate resources required for scaling up contracting. This study attempts to fill this critical knowledge gap by analyzing costs of contracted out health facilities for MNH services in two remote rural districts of Pakistan. In Pakistan, contracting out was piloted for Basic Health Units (BHUs) in 2003 and scaled up in 2008 to BHUs of all provinces in the country [7, 8]. The most recent initiative includes contracting out of the next level of care facility i.e. Rural Health Centers (RHCs) for MNH services in selected districts. The objective of this study was to ascertain unit costs and distribution of expenditures at contracted out RHCs in remote rural settings for the actual volumes of MNH services provided in year 2011, and for the estimated higher volumes of services needed by the catchment population. The knowledge generated through this study will enable policy makers to develop optimal resource envelopes and set performance targets for contracting out MNH services in order to accelerate progress towards achieving Millennium Development Goals (MDGs) 4 and 5.

### Setting

Rural Health Centers (RHCs) are frontline facilities typically providing Primary Health Care (PHC) and limited in-patient care including MNH services. The two RHCs in this study are located in remote rural locations in the two provinces of Sindh and Khyber Pukhtunkhwa and serve small, dispersed populations with limited road transport. These RHCs had been contracted out to a national NGO since 2008 and each contractual package involved the provision of MNH services. These RHCs were selected for this study because at the time they were the only contracted out RHCs in Pakistan and the NGO operating them was able to provide accurate financial, staffing and service provision data required for this costing study. The contracts did not specify targets for an agreed service package and were based on block grants. The controls provided by these contractual arrangements to the NGO included: authority over existing government staff with no power to transfer or terminate; hiring of additional staff with higher salaries supported by NGO; authority over maintenance of building and equipment; introduction of extended categories of essential drugs and diagnostics; and introduction of user fees for antenatal and delivery registration and additional diagnostics that were not covered by the RHC budget.

Keti-Bunder RHC is remotely located in Thatta District of Sindh province with a primary catchment population of 14,004. A national NGO was contracted to provide the same package of services mandated at all RHCs including routine MNH services and Basic Emergency Obstetric and Neonatal Care (BEmONC) services. There are no other facilities providing MNH services in the catchment area. Patients needing higher level MNH care including Comprehensive Emergency Obstetric and Neonatal Care (CEmONC) services were directed to the nearest referral facility, a government hospital approximately two hours travel by automobile. The maternal health services at Keti-Bunder are provided by a midwife and staff nurse with minimal laboratory tests and a limited medicine formulary. The medical officer routinely provides newborn examination and essential treatment as required.

The Shagram RHC is located in Chitral District in the province of Khyber Pukhtunkhwa with a primary catchment population of 16,039. It was contracted to the same national NGO, but tasked to provide all RHC mandated services as well as Comprehensive Emergency Obstetric and Newborn Care (CEmONC) services including C-Section and assisted deliveries, which are beyond the BEmONC services normally provided by RHCs. This is because travel to the next closest facility offering CEmONC services required 2–3 hours by automobile on poor roads in mountainous terrain, and for almost half of the year access is impossible due to weather conditions. Also, in the Shagram primary catchment area there are no other health facilities or private medical practitioners, thus provision of CEmONC services at Shagram RHC is critical for saving the lives of mothers and newborns. The MNH services at Shagram RHC are routinely provided by an obstetrician and team of Lady Health Visitors (LHVs) and staff nurse/anesthetists using a wide range of laboratory tests and medicines.

## Methods

This study is part of a larger project assessing comparative effectiveness of contracted out versus non-contracted RHCs for improving access to MNH services [9]. A case study design is applied with an in-depth study of provider costs at the two contracted out RHCs. Data was collected between April to June, 2012, on all annual cost inputs and patient volumes for 2011. The MNH services costed were Antenatal Care (ANC) Visits, Post Natal Care (PNC) Visits, Normal Delivery, Assisted Delivery, C-Sections and Newborn Care inclusive of well baby check-up, screening test for jaundice, BCG immunization and treatment for infection.

### Costs for actual and projected volumes

Costs were calculated based on the actual costs of inputs and the volumes of MNH services provided in 2011 regardless of whether these were optimal levels of inputs for the actual volumes. Costs were also calculated for the projected volume of services needed by the catchment population of each RHC at the optimal utilization of inputs. The projected volume of ANC visits, deliveries, PNC visits, and newborn care was determined based on the assumption that all women would be utilizing the RHC as there were no other skilled MNH service providers in the catchment area. Information on the percentage of women of reproductive age and general fertility rate for rural populations from the Pakistan Demographic and Health Survey (PDHS) 2006–07 was used to calculate the number of pregnant women in each RHC catchment area population. For information on how many pregnant women would require assisted deliveries and C-sections we used the estimates provided by hospital based studies in Pakistan serving mostly rural populations [10–12]. These estimates were used to calculate projected volumes of MNH services needed in each catchment population. Standard Treatment Guidelines and information from the technical staff and external medical specialists were used to determine the technical staff times, the medicines and the medical, laboratory and ultrasound supplies required to deliver each MNH service.

### Variable and fixed costs

Variable costs included technical staff salaries (e.g. doctors, nurses, pharmacists) medicines and medical, laboratory and ultrasound supplies used for MNH services. Fixed costs included administrative and support staff salaries, and other fixed operating costs including utilities, stationary, repairs and maintenance, generator fuel and depreciation of capital assets.

### Data collection

National Standard Treatment Guidelines (STG) obtained from the Pakistan Maternal and Newborn Child Health Program were used to calculate inputs required for each MNH service. Actual staffing levels were assessed through RHC records and direct observation during field visits by the study team. The study team also undertook in-depth interviews of provider staff using a template developed in consultation with a consultant pediatrician and obstetrician to collect data on the time used to provide each MNH service. Records and inventory of the contracted Rural Health Centers, and financial and indent records of the NGO contracted to manage these RHCs were reviewed to obtain actual cost information on supplies, equipment, utilities and other operational costs. Since staff salaries differed at each RHC, the salary costs were taken as the current mid-range salaries in each facility. Unit costs for each medicine were taken as the median cost of all the generic versions available in Pakistan based on the current Standard Pharmaguide without adjustment for discounts. Unit costs for vaccines were based on cost information obtained from Ministry of Health Central Office of the Expanded Program of Immunization (EPI).

### Cost analysis

While there are various methods available to analyse costs of healthcare services, a systematic review concludes that there is no single best method for all type of services and hence the most suitable method can be adapted according to the study context [13]. After this review in 2005, a team at Management Sciences for Health in Boston, USA, developed the CORE Plus costing tool in 2008 to improve on existing methodologies. ^a^This study used the CORE Plus costing tool to calculate and analyse the distribution of MNH service costs, the costs per service and the minimum staffing levels for each RHC for the 2011 actual service volumes and for the volumes projected to meet the needs of the catchment populations. The CORE plus tool is a bottom-up, micro-costing method using the Microsoft Excel platform and is specifically adapted for integrated community and facility based PHC and first level referral hospital services. It requires detailed cost, staffing, activity volumes and catchment population data for each facility which is well suited to a case study design. It is also suitable for and has been used more widely for multi-facility studies. Unlike other methods, it explicitly incorporates Standard Treatment Guidelines (STG) based quality standards into its costing calculation, and calculates the costs of the volumes of services required by a facility’s catchment population. It is also policy friendly because it provides the detailed cost information needed to develop appropriate resource envelopes and performance targets for contracting out health services.

The following categories of information were entered into the CORE Plus Costing tool: demographic and epidemiological data for the catchment population; the type and volume of services provided; staff numbers, categories, salaries, working hours, holidays, proportion of administrative support time; medication, vaccination, medical and diagnostic supplies; and other operating costs. Although direct capital expenditures are not included in the CORE Plus costing tool, annual depreciation was entered so that provision for replacement capital costs is included. This tool has been widely used to analyze costs for provision of all primary health care services, including contracted out services, delivered in the community and at health posts and basic and comprehensive health centers [14–16]. The Core Plus tool distributes indirect costs proportionally to each MNH service based on the proportion of total direct MNH provider staff costs used to provide each service.

### Ethical approval

Ethical clearance for the interviews and record review was obtained from the Ethics Review Committee of the Aga Khan University as part of the larger study [9]. Two electronic databases used for this study were open access: Pakistan Demographic and Health Survey (PDHS) 2006–07; Standard Pharmaguide for Pakistan. Access to the medications and supplies price list electronic database for the two health facilities was provided by the non-governmental organization managing these facilities with the permission of the CEO, as were the other health facility cost and activity data which were provided in hard copy. Informed verbal consent was obtained from the health facility staff interviewed, all information collected was kept anonymous and all procurement and indent records were confidentially treated.

## Results

### Average costs per MNH service

The average cost per MNH service for the actual volumes of contracted out services provided in 2011 by the BEmONC RHC was US$ 36.45, while that of the CEmONC RHC was US$ 64.65 (see Table 1). The average cost per MNH service for the projected volume of services needed was US$ 23.05 at the BEmONC RHC and was US$ 40.97 at the CEmONC RHC. The average cost for the actual volumes is based on the actual mix of MNH services provided, whereas the average cost for the projected volumes is based on the service mix according to the package of MNH services to be received by every mother according to the MNCH national treatment guidelines, which incorporate the WHO recommended package of MNH services [17]. Tables 2 and 3 provide the cost for each type of MNH service at actual and projected volumes needed for each RHC. At both RHCs the average cost of each MNH service for projected volumes is lower than that for actual volumes.

**Table 1:** **Distribution of average costs per MNH service at the actual and projected volumes**

	BEmONC RHC				CEmONC RHC			
Distribution of costs	Actual volumes		Projected volumes		Actual volumes		Projected volumes	
	US$ *	%	US$ *	%	US$ *	%	US$ *	%
Salaries (Technical staff)	14.35	39.4	10.57	45.9	29.47	45.6	22.87	55.8
Salaries (Admin and support staff)	9.46	26.0	4.99	21.7	19.27	29.8	9.73	23.8
Medicines, medical, laboratory & ultrasound supplies	3.87	10.6	4.00	17.3	4.28	6.6	4.48	10.9
Other operating costs	8.77	24.0	3.49	15.1	11.63	18.0	3.89	9.5
***Average cost per MNH Service***:	36.45		23.05		64.65		40.97	

*1US$ = PKR 86.02 (average rate for Year 2011).

**Table 2:** **BEmONC RHC cost per service**

MNH services	Actual volume of services in 2011	Cost based on actual volume US$**	Projected volume of services needed	Cost based on projected Volume US$**
Antenatal care visit	892	18.78	1968	13.39
Normal delivery	108	84.61	492	56.17
Postnatal care (PNC) visit	123	13.86	200	9.73
Community PNC visit	141	20.89	292	12.71
Newborn care*	108	16.86	492	10.59

*Includes BCG & first Polio vaccinations.

**1US$ = PKR 86.02 (average rate for Year 2011).

**Table 3:** **CEmONC RHC cost per service**

MNH services	Actual volume of services in 2011	Cost based on actual volume US$**	Projected volume of services needed	Cost based on projected volume US$**
Antenatal care visit	824	45.50	2,254	31.09
Normal delivery	210	148.43	437	98.65
Postnatal care visit	48	27.34	564	18.68
Newborn care*	226	41.07	564	22.20
Assisted delivery	10	167.43	42	100.07
Caesarean section	6	183.34	85	129.98

*Includes BCG & first Polio vaccinations.

**1US$ = PKR 86.02 (average rate for Year 2011).

### Expenditure distribution

The breakdown of costs based on actual volumes at the BEmONC RHC was: direct care salaries 39.4%, medicines and medical and diagnostic supplies 10.6%, administrative and support salaries 26%, and other operating costs 24%. At the CEmONC RHC the breakdown was: direct care salaries 45.6%, medicines and supplies 6.6%, administrative and support salaries 29.8%, other operating costs 18% (see Table 1). At projected volumes the proportion of variable costs increased by 26% and 28% at the BEmONC and CEmONC RHC respectively, and the proportion of fixed costs decreased by 25.5% and 30% respectively.

### Unit costs of MNH services

The costs per service based on actual volumes at the BEmONC RHC were: antenatal care (ANC) visit USD$ 18.78, normal delivery US$ 84.61, newborn care visit US$ 16.86 a postnatal care (PNC) visit US$ 13.86, and a community PNC visit US$ 20.89 (See Table 2). At the CEmONC RHC these costs were: ANC visit US$ 45.50, Normal Delivery US$ 148.43, assisted delivery US$ 167.43, C-section US$ 183.34, Newborn Care visits US$ 41.07 and PNC visit US$ 27.34 (see Table 3).

When the volume of MNH services is increased from the actual 2011 level to meet the estimated needs of the catchment population, the cost for each MNH service at both RHCs is reduced. For different MNH services these reductions ranged from 29-39% at the BEmONC RHC, and from 29-46% at the CEmONC RHC^b^.

### Minimum service provider staff levels

The existing number of provider staff at both RHCs was captured and additionally we calculated the minimum provider staff strength required for quality delivery of the actual and the projected volumes of MNH services needed (see Table 4). The minimum amount of provider staff time required, measured in Full Time Equivalents (FTEs), was calculated based on the provider staff times needed to meet STG standards for each MNH service.

**Table 4:** **Full Time Equivalents** (**FTEs**) **for 2011 workload and for projected volume of MNH services**

	BEmONC RHC			CEmONC RHC		
Technical staff	Existing FTEs for actual volumes	Minimum FTEs required for actual volumes	Minimum FTEs required for projected volumes	Existing FTEs for actual volumes	Minimum FTEs required for actual volumes	Minimum FTEs required for projected volumes
Medical officer	0.10	0.04	0.16	0.04	0.06	0.14
Staff nurse	1.00	1.51	4.58	-	-	-
Staff nurse/Anaesthetist	-	-	-	2.00	1.62	4.05
Lady health visitor	-	-	-	3.00	1.67	4.35
Midwife	1.00	1.79	5.49	-	-	-
Obstetrician	-	-	-	1.00	0.81	2.32
Lab technician	-	-	-	1.43	0.51	1.36
Dispenser	0.50	0.33	0.77	0.68	0.31	0.92
Vaccinator	0.40	0.03	0.11	0.03	0.05	0.12
***Total FTEs***	3.00	3.70	11.11	8.18	5.03	13.26

A comparison of the existing with the minimum required provider staff for actual 2011 volumes shows that the medical officer, dispenser, and vaccinator are present at facility but underutilized for MNH services in the BEmONC RHC, while there is shortage of staff nurse and midwife time. However, this comparison in CEmONC RHC shows that there is underutilization of the obstetrician, staff nurse/anaesthetist, lady health visitor, lab technician and dispenser, and a shortage of medical officer and vaccinator time.

Staff productivity increases when the provider staff increases to the minimum staffing levels required for the projected volume of MNH services. At both RHCs the average number of services provided per staff hour increases and the provider staff salary cost per MNH service decreases for each MNH service.

## Discussion

This study analyzed provider costs of two contracted out health facilities in remote rural locations for delivering CEmONC and BEmONC services in a developing country setting, whereas past studies have reported costs of selected maternal health services in government managed facilities. It reports the provider cost per MNH service in 2011 and the provider expenditure distribution per MNH service for actual service volumes and for higher volumes required to meet the needs of the total service population for MNH services. The study raises a number of salient findings discussed below.

In developing countries such as Pakistan, staff costs at government RHCs consume the major share of cost and are as high as 78% of total budget [18]. This study found the personnel costs at the BemONC RHC, whose service package is identical to that of government managed RHCs, to be the most significant share of total cost at 66%. This is lower than the comparable proportion at government RHCs which may be due in part to higher utilization than at government RHCs, thus proportionately higher medicine and supply costs and lower personnel costs. The few studies available from other developing countries on MNH costs of government managed health centers providing BemONC level care report a lower proportion of personnel costs ranging from 39% to 45% [15, 19]. A factor contributing to this difference could be the smaller, widely dispersed service populations served by the two study RHCs resulting in lower utilization, and consequently proportionately lower costs of medicines and supplies and higher personnel costs. The proportion of personnel cost at the CemONC RHC is understandably higher at 75% of actual costs due to deployment of higher staff numbers and skill mix in line with the higher acuity care provided. Within personnel costs, the study differentiated the cost of technical versus administrative personnel. Our study found the cost of technical personnel was 60% of total salary costs at both contracted RHCs, however no comparable data is available.

Our results show a difference in total costs and costs per MNH service between the two RHCs. This is not surprising because the CEmONC RHC is staffed, equipped and supplied to provide a full range of CEmONC services with a higher cost staff skill mix and numbers, while the other RHC provides selected BEmONC services requiring a less intensive level of inputs with lower costs.

The unit costs of an ANC visit and a normal delivery at the contracted out RHCs in our study fall within the ranges reported in developing countries. The ANC cost per visit reported in Ghana for basic health care centers in 2010 ranged from US$ 0.99 – 49.17 [19]. The BEmONC RHC cost of US$ 18.78 for an ANC visit is about midrange and the CEmONC RHC cost of US$ 45.50 is at the higher end of the range. The cost of a normal delivery in Ghana study ranged from US$ 12.70 -152 [19], and US$ 40–105 at hospitals in other developing countries [3, 20, 21]. The cost of a C-section delivery in these hospital studies has been reported to be 2–5 times higher than a normal delivery [3, 20, 21]. Again the BEmONC RHC cost for a normal delivery of US$ 84.61 is about midrange, and the CEmONC RHC cost of US$ 148.43 is at the higher end of the range reported for Ghana. The unit cost of US$ 183.34 for a C-Section provided at CEmONC RHC is much lower than 2–5 times the cost of a normal delivery, which is well below the range of C-Section costs reported by the hospital studies cited above [3, 20, 21]. However, the higher cost staff skill mix at the at the CEmONC RHC puts the costs for normal delivery and ANC visit at higher end of the range reported for Ghana. Hence, there is a cost trade-off in providing CEmONC services at an RHC.

The study also showed that the projected volume of MNH services needed by the primary catchment populations of each RHC in this study exceeded that provided at either RHC in 2011 despite the absence of other skilled MNH service providers in either catchment area. This relatively low volume of service demand is a dilemma faced by health facilities serving smaller, remote and more dispersed low income populations as reported in a multi-country study where the majority of PHC facilities were found to be under-utilized and under-resourced especially in terms of staffing [22]. Given their actual MNH service staffing and workloads, both study RHCs were underutilized and the BEmONC RHC was understaffed.

Although higher service volumes increase the total costs for provider staff, medicines, diagnostic tests and supplies, the unit costs for these do not change, while the administrative and support salaries and other operating costs stay fixed resulting in a lower cost per service for each MNH service. Increases in volumes of these services also result in increased staff productivity due to improved staff utilization which further reduces the cost per service. At the BEmONC RHC the cost of per service for an ANC visit and for a normal delivery is reduced by 28.7% and 35.5% respectively, while these costs at the CEmONC RHC are reduced by 31.7% and 33.5%. Evidence reports that increasing the volumes of ANC visits and normal deliveries by 10% reduces the cost per service by 32% and 18.75% respectively [19].

Contracting out is essentially a supply side initiative. Previous papers report higher service utilization of contracted out PHC facilities due to improved functioning of these facilities and provision of adequate levels of staffing, medicines, supplies and equipment [7, 8, 23–26]. It is also reported that the middle income population bracket utilize these contracted out facilities more than the lower income bracket due to a range of barriers. Evidence based resource envelopes and results based performance targets for contracting out MNH services can be developed based on the unit costs and minimum staffing levels calculated for the needed service volumes. These volumes would be based on the projected MNH service needs of the proportion of the catchment population that should be able to access services at the facility. Both financial and non-financial barriers, especially for the poorer population, need to be effectively addressed if the utilization and unit costs of contracted out facilities are to achieve the performance targets within their resource envelopes.

### Strengths and limitations

This study provides detailed cost data for MNH services and there is dearth of such data from developing countries. In addition to calculating costs for actual MNH service volumes, this study determined costs for delivering the projected volumes of MNH services needed by the catchment populations through the two contracted out facilities. This is the information required for developing results based performance targets and evidence based resource envelopes sufficient for provision of quality MNH services.

There are a few limitations of the study. The estimated number of any particular MNH service needed by the catchment populations used to calculate service costs may be over stated as we assumed all services would be provided in the RHC, which is not practically possible. Hence, the costs per service for the lower volumes may be higher.

Information on the technical staff times needed to provide each MNH service was collected from the staff providing these services. Developing these technical staff times based on a consensus of clinical experts in the delivery of rural health services would reduce the staff self-reporting bias inherent in this study.

Although alternative funding mechanisms, including contracting out through public private partnerships, are being introduced in Pakistan to increase access to health services, the assessment of these health sector reforms is beyond the scope of this paper. The results of our study can assist the government to develop evidence based performance targets for contracted out MNH services regardless of how the costs are shared between public and non-government funding.

Road transport, particularly in bad weather conditions, is a key factor limiting access to the study RHCs. However, to estimate the proportion of the population needing MNH services that would not be able to access each these facilities due to weather conditions, and to estimate the consequent reduction in MNH services needed to be delivered by each RHC, was not possible within the limitations of this study. As there were no other skilled MNH service providers operating in the primary catchment areas of these facilities, our projected volumes of MNH services are based on meeting the needs of all mothers and newborns in the catchment populations.

### Future areas of research

While this was an in-depth case study, future studies should include a larger pool of facilities. Moreover, costing of government managed along with contracted out RHCs is required to provide meaningful comparisons.

## Conclusions

Despite the lack of comparable data in Pakistan, by contracting out government rural health facilities in remote settings, the unit costs of MNH services have remained higher than is optimal, primarily due to low utilization. Contracting out health services has been reported to increase utilization of PHC facilities. Policy measures such as results based performance targets need to be developed to track the utilization and efficiency of contracted out services and to measure whether they are meeting the needs of the population. Provider cost analysis using STG based service costing frameworks should be applied across a number of health facilities to guide development of evidence based resource envelopes. This study provides a starting point for further costing studies and development of evidence based resource envelopes and results based performance targets for contracting out.

## Endnotes

^a^The WHO website has a webpage of costing tools, including the Core Plus tool, each of which has been independently reviewed to assess technical validity: http://www.who.int/pmnch/knowledge/publications/costing_tools/en/.

^b^A more detailed breakdown of average costs for each MNH service can be provided on request.
